# Supplementary material for: Comprehensive Annotation and Functional Exploration of MicroRNAs in Lettuce
Source: Front Plant Sci. 2021 Dec 24;12:781836. doi: 10.3389/fpls.2021.781836 (PMC8739914; doi:10.3389/fpls.2021.781836)
Supplement: Supplementary file 2 [file Data_Sheet_8.ZIP › GSM2230750.plot/Lsa-miRN34_Lsat_1_v5_gn_2_54221.1_1164_TPlot.pdf]

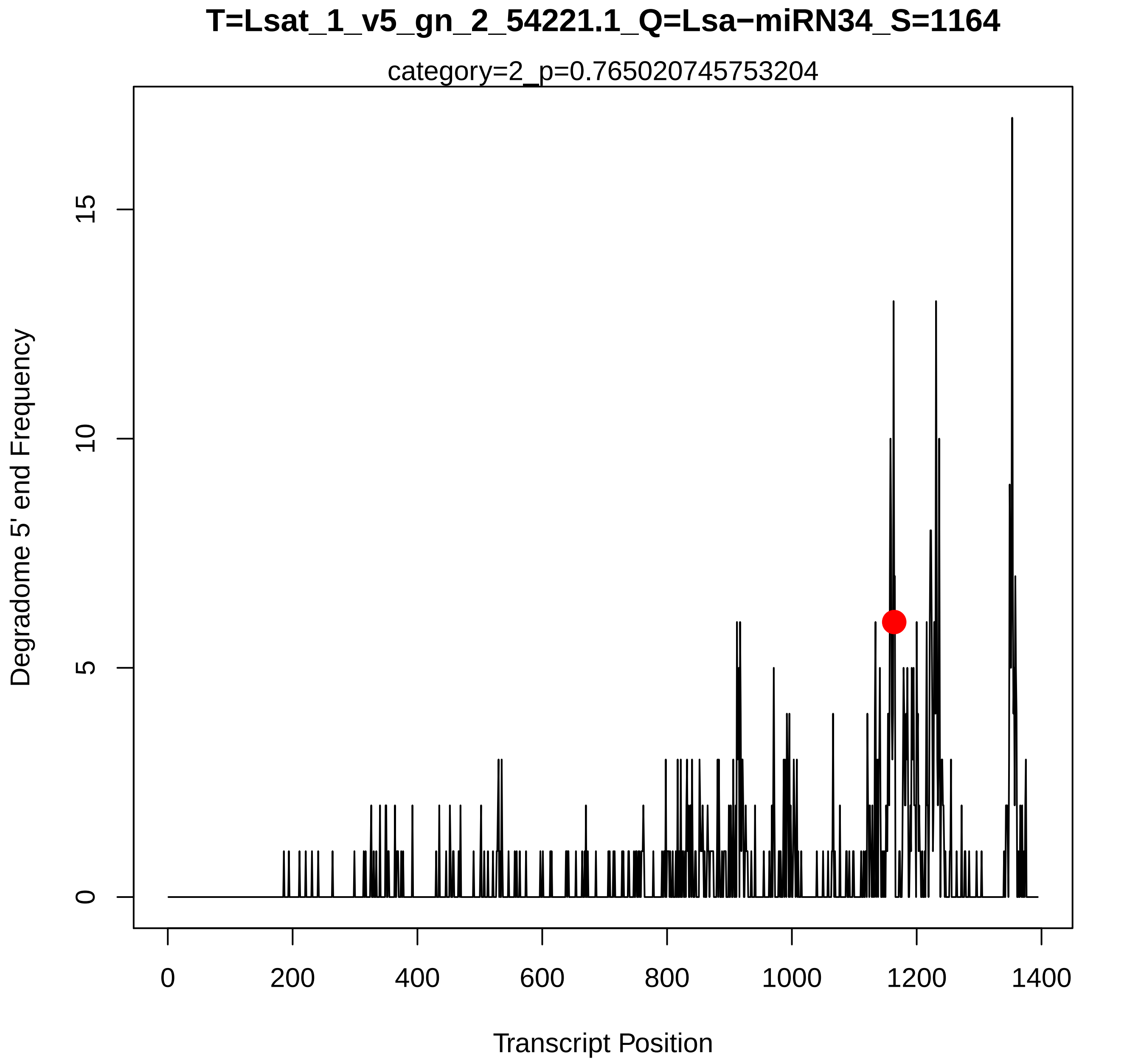
T=Lsat_1_v5_gn_2_54221.1_Q=Lsa−miRN34_S=1164
category=2_p=0.765020745753204
Degradome 5' end Frequency
0
5
10
15
0
200
400
600
800
1000
1200
1400
Transcript Position
